# Evaluating the effect of non-invasive force feedback on prosthetic grasp force modulation in participants with and without limb loss

**DOI:** 10.1371/journal.pone.0285081

**Published:** 2023-05-04

**Authors:** Federica Barontini, Meegan Van Straaten, Manuel G. Catalano, Andrew Thoreson, Cesar Lopez, Ryan Lennon, Matteo Bianchi, Karen Andrews, Marco Santello, Antonio Bicchi, Kristin Zhao

**Affiliations:** 1 Department of Soft Robotics for Human Cooperation and Rehabilitation, Istituto Italiano di Tecnologia, Genoa, Italy; 2 Department of Physical Medicine & Rehabilitation, Mayo Clinic, Rochester, Minnesota, United States of America; 3 Department of Quantitative Health Sciences, Mayo Clinic, Rochester, Minnesota, United States of America; 4 Department of Information Engineering, University of Pisa, Pisa, Italy; 5 Research Center “E. Piaggio”, University of Pisa, Pisa, Italy; 6 School of Biological and Health Systems Engineering, Arizona State University, Tempe, Arizona, United States of America; University of Illinois at Urbana-Champaign, UNITED STATES

## Abstract

Grasping an object is one of the most common and complex actions performed by humans. The human brain can adapt and update the grasp dynamics through information received from sensory feedback. Prosthetic hands can assist with the mechanical performance of grasping, however currently commercially available prostheses do not address the disruption of the sensory feedback loop. Providing feedback about a prosthetic hand’s grasp force magnitude is a top priority for those with limb loss. This study tested a wearable haptic system, i.e., the Clenching Upper-Limb Force Feedback device (CUFF), which was integrated with a novel robotic hand (The SoftHand Pro). The SoftHand Pro was controlled with myoelectrics of the forearm muscles. Five participants with limb loss and nineteen able-bodied participants completed a constrained grasping task (with and without feedback) which required modulation of the grasp to reach a target force. This task was performed while depriving participants of incidental sensory sources (vision and hearing were significantly limited with glasses and headphones). The data were analyzed with Functional Principal Component Analysis (fPCA). CUFF feedback improved grasp precision for participants with limb loss who typically use body-powered prostheses as well as a sub-set of able-bodied participants. Further testing, that is more functional and allows participants to use all sensory sources, is needed to determine if CUFF feedback can accelerate mastery of myoelectric control or would benefit specific patient sub-groups.

## Introduction

Limb loss is a condition that affects millions of people. Globally, 57.7 million people lived with traumatic limb loss in 2017 [1], with the prevalence of amputation due to trauma/cancer in the United States alone expected to reach 1.35 million by 2050 [2]. It is estimated that thirty percent of current people with amputation have lost an upper limb [3]. That includes the hand, forearm, and upper arm.

Upper limb loss can be especially life altering due to the dependence on bimanual operations for many activities of daily living, such as recreational activities and sports. Despite recent efforts to augment the lost motor function of the hand through active prostheses [4], discontinued or reduced use of prostheses continues at high rates [5, 6]. The lack of proper restoration of touch-related sensory feedback is a severe drawback and may play a significant role in the reason for rejection of a prosthesis [5]. In addition, a residual limb with intact sensation often reveals itself to be more functional than an insensate prosthesis [7]. Thus, sensation plays an important role in integration and embodiment of a prosthetic arm for the user.

The current research to address loss of sensation in individuals with limb loss consists of the restoration of the haptic channel through invasive and non-invasive techniques. Invasive feedback can be provided with electrodes implanted surgically, relaying information directly to the nervous system [8, 9]. Non-invasive techniques rely on wearable haptic feedback devices. The latter solutions, referred to as *supplementary feedback* in this paper, convey touch-mediated information through skin-stretch [10], pressure [11, 12], vibration or electrical stimulation [13, 14].

Several types of information can be delivered through a supplementary feedback loop, including grip/grasp force, hand aperture, and contact [15]. Survey results of upper limb prosthesis users suggest that a top priority for input in the feedback loop is the prosthetic hand’s grip force magnitude [16–18]. Prosthesis users worry about over-squeezing and hurting others or breaking fragile objects [19]. Previous studies have investigated modulation of grasp force using vibrotactile, electrotactile, mechano-tactile, and augmented reality feedback [20, 21]. In a thorough review of these studies by Stephens-Fripp, et al. [20] that identify current disadvantages to these feedback mechanisms and opportunities to bring effective supplementary feedback closer to fruition, the authors discuss *the need for different testing approaches and feedback mechanisms for each type of prosthetic hand*, *such as the novel* SoftHand Pro (SHP). The unique compliant grasp of the SHP differs significantly from the rigid prostheses that have been previously used in evaluating artificial feedback, and *it is currently unknown whether conventional feedback approaches perform similarly in a prosthesis with a compliant grasp*.

The SHP is a robust and functional myoelectric prosthesis that minimizes cost and weight with an underactuated design of a single motor and soft adaptable robotics technologies [21]. Functionally, this design is unique because of its synergistic movement pattern that adapts to the shape of the object it is grasping. The SHP offers a versatile platform into which a novel haptic device can be integrated. Therefore, an upper arm squeezing force feedback prototype was designed to communicate directly with the SHP platform. The Clenching Upper-Limb Force Feedback Device (CUFF) [22] provides force feedback stimuli similar to the sensation of getting one’s upper arm squeezed by a blood pressure cuff. Ajoudani et al. [23], tested the CUFF with able-bodied participants in a manufacturing setting and found an improvement in tele-operation force control. In another study [24], the CUFF was used with the SHP to investigate grasp force while lifting an object in two different conditions (with and without the force feedback). The results did not show any significant differences between the two conditions; however, the study was only performed on a small cohort of able-bodied participants, and the CUFF’s motor control algorithm was further optimized. Specifically, the original CUFF’s motors were proportionally mapped to the closure of the SHP, while the current CUFF has a logarithmic relationship between the hand closure and the rotation of the CUFF’s motors. This change was made because in a previous study participants desired more feedback sensitivity during larger grasp forces when the SHP would be at risk of over squeezing a fragile object. This study designed a new task that required force modulation and tested whether the use of the CUFF improved participants’ ability to modulate the grasp force of the SHP. Our hypothesis was that the participant’s grasping motion of the robotic hand, during the target force task, would be more precise with the CUFF than without and more similar to the sound limb.

## Materials

### The SoftHand Pro

The SHP [25] is a myoelectric prosthetic version of the Pisa/IIT SoftHand. The SHP is designed and made by the authors team. Some components of the SHP are bought from qbrobotics s.r.l., a company producing robotic hands and an industrial version of the Pisa/IIT SoftHand. The Pisa/IIT SoftHand combined compliance and synergy inspiration [26, 27] into an artificial soft hand with 19 degrees of freedom (DoFs), 4 on each finger, and 3 on the thumb. The fingers are capable of flexion/extension as well as ab/adduction. The hand is actuated by a single DC motor (15-Watt Maxon DCX 22S), and a gearhead of 86:1. With this setup, the hand can provide up to 76 N of force in power grasp and 20 N in pinch grasp.

**Fig 1:**
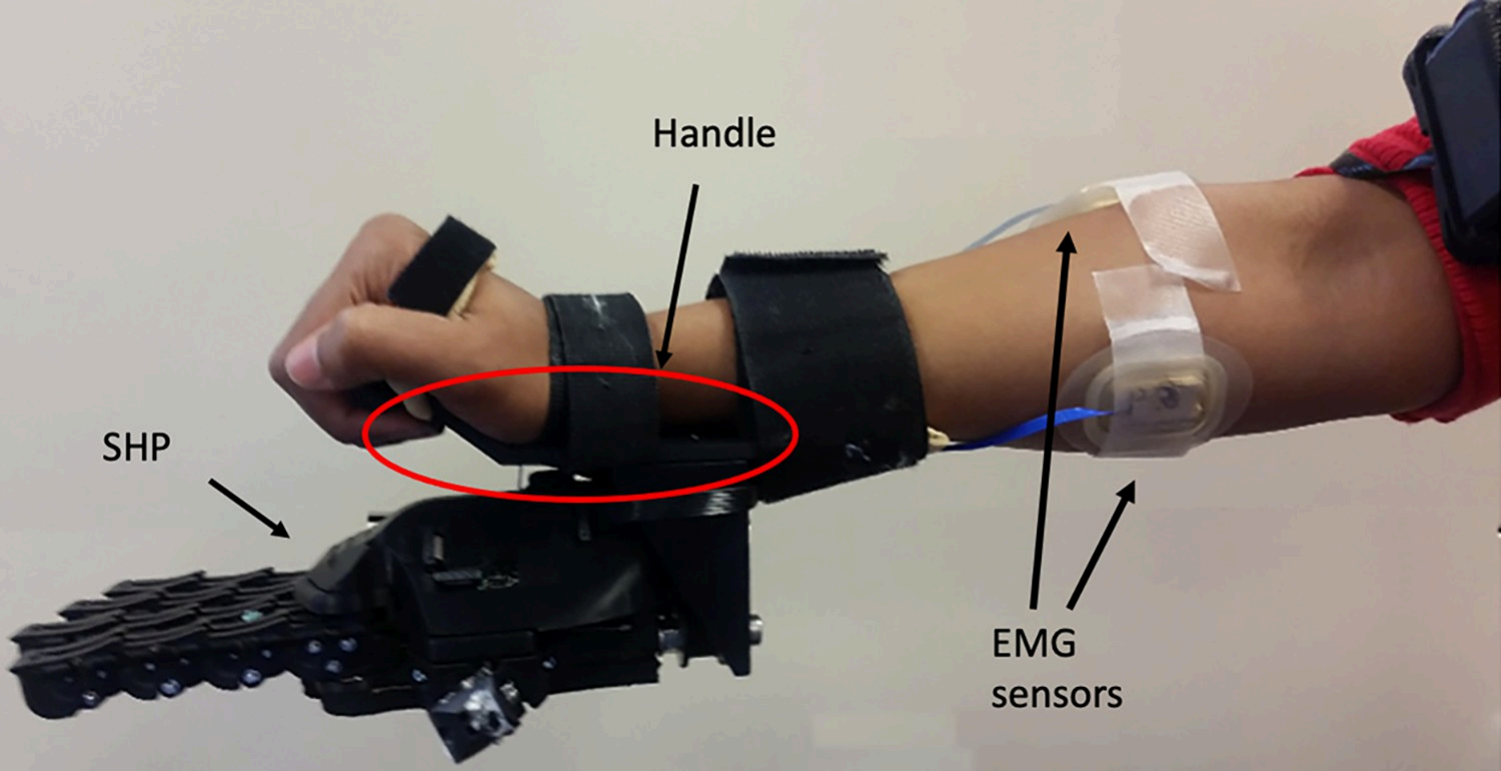
The 3D printed orthosis. The picture shows the SHP attached to a forearm adapter used by the able-bodied participants.

Further information about the SHP is published on the Natural Machine Motion Initiative website (http://www.naturalmachinemotioninitiative.net/) [28]. The SHP can easily adapt to the environment as all of the fingers conform to the shape of grasped objects which augments its grasping capabilities. In addition, the SHP interfaces with commercially available surface electromyography (EMG) sensors (Otto Bock, Germany) to allow control of the hand. EMG sensors were placed on the flexors and extensors of the forearm to activate closing and opening of the SHP, respectively. Proportional myoelectric control of the SHP used a FCFS (first come first serve) approach which acts on (by opening or closing) the first electrode signal to go above a minimum threshold and is controlled by only that signal until it drops below threshold [21]. For the cohort with limb loss, the SHP was connected to the participant’s personal socket through a commercial wrist (Quick Disconnect Wrist, Ottobock, Germany), or a custom molded socket fabricated for an earlier SHP research study [25]. For the able-bodied participants, the SHP was mounted on a one size fits all 3D printed orthosis (similar to a wrist cock up orthosis) that was secured on the user’s forearm with a Velcro band (Fig 1).

### The force feedback CUFF

**Fig 2:**
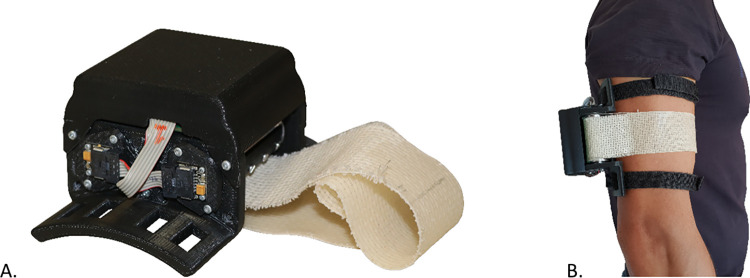
The Clenching Upper-Limb Force Feedback (CUFF). A) On the left the CUFF device, and B) a user wears the CUFF on their arm.

As mentioned previously, the force feedback device used in this study is a reengineered version of the Clenching Upper-Limb Force Feedback (CUFF) [22]. The redesign included a mechanical reduction of the device in terms of weight and dimension, and a different strategy for the motor control. Briefly, the CUFF device consists of a primary frame, which can be secured to the user’s arm with two VELCRO straps, an actuation unit composed of two Maxon DC motors, powered by a 12 V battery, with a gearbox ratio of 64:1, and a belt used as an interface surface with the human body (Fig 2). The wearable device can provide distributed mechano-tactile stimulation to the upper arm because the motors are programmed to rotate in opposite directions, tightening or loosening the band and increasing or decreasing the pressure on the arm. The user is instructed to associate the stimulus from the CUFF with the grip force of the SHP. To estimate the grip force exerted by the hand, the residual current (*RC_meas_*) was calculated. The residual current is described as the difference between the estimated current and the real current absorbed by the motor of the SHP. For further detail see [29]. Mapping the information from the robotic hand into the feedback device requires an association between each value of the hand with a corresponding position value of the CUFF motor. A non-linear mapping was chosen to define the reference position (*pos_Cref_*) of the motors. Two constraints were defined to identify the non-linear relation: when *RC_meas_* = 0 also the *pos_Cref_* = 0; and when ${RC}_{meas}=\frac{2}{3}{RC}_{max},\;{pos}_{Cref}=\frac{1}{3}{pos}_{Cmax}$. So, the new reference position of the motors will be:

$${pos}_{Cref}=\beta log\left(1-\alpha \frac{{RC}_{meas}}{{RC}_{max}}\right){pos}_{Cmax}$$

The constraints were imposed by choosing the correct value for the constants: *α* = 0.9510, and *β* = −0.3317. The values of the constant were extracted using the non-linear models provided by the Curve Fitting Toolbox in MATLAB. *Cmax* position was set at 800 ticks from the zero position as a result of testing that determined this position to be the maximum position well tolerated by a group of individuals.

### Instrumented cylinder

As a means to assess the grip forces generated by the SHP, a cylindrical object (height 12 cm and diameter 7.5 cm) consisting of two hemicylindrical blades, was fabricated using 3D printing technology and each blade was instrumented with a 6-axis load cell (Nano 25, ATI Industrial Automation, Apex, NC, USA). Load cell data from this instrumented cylinder was collected with a 12 channel Analog/Digital board with USB communication (Model 8012, National Instruments, Austin, TX, USA) and sampled at 100 Hz using a simple control program (LabVIEW, National Instruments). Data were analyzed with a MATLAB script file (MATLAB 2019a, MathWorks Inc., Natick, MA, USA).

## Methods

### Research participants and study schedule

Two cohorts participated in this study. The first cohort was comprised of five participants with trans-radial limb loss (mean age 43.20 ± 16.79 years, 4 males/1 female, Table 1). The second cohort was comprised of 19 able-bodied participants (8 males, mean age 39.75 ± 8.87 years; 11 females, mean age 32 ± 11.79 years). The sample size was not determined from a power analysis. The number of participants in each cohort was the maximum number of participants that could be recruited and participate in data collection in the three-month period that was dedicated to this aspect of the project. This sample size also aligned with other prosthetic studies of these populations. Both groups were tested at Mayo Clinic in Rochester (MN, USA) using the SHP in conjunction with the CUFF.

**Table 1:** Demographics of the participants with limb loss.

Participant	Age at time of testing (yrs)	Years Since Amputation (at time of study)	Side of Limb Loss	Previous Hand Dominance	Gender	Participant owned Prosthesis Type
1	31	7	L	R	M	BP* Hook
2	31	18	R	L	M	BP Hook
3	67	24	R	Unknown	F	Tridigit MP*
4	32	3	L	R	M	BP Hook
5	55	2	R	R	M	Multigrasp MP

*BP indicates body-powered, and MP indicates myoelectric prosthesis.

Eligibility criteria for the cohort with limb loss included individuals with a history of elbow disarticulation, trans-radial or trans-humeral limb loss ≥ 6 months prior to study enrollment, aged ≥ 18 years, and no prior experience utilizing the CUFF or any other force feedback device. Exclusion criteria included a clinical history of brachial plexopathy, cervical radiculopathy or polyneuropathy, orthopedic or joint impairments that would severely limit upper limb function, central nervous system disease limiting upper limb function, significant rigidity of the upper limb, medication use that might limit sensory or motor functions, and psychiatric or cognitive limitations that would prevent the ability to perform the study tasks.

For the cohort with limb loss, familiarization and data collection occurred over two consecutive days. The first day was dedicated to the familiarization with the SHP and the feedback CUFF as well as collection of two clinical assessments, the results of which are not reported in this work (Activities Measure for Upper Limb Amputee (AM-ULA), and the Jebsen Taylor Hand Function Test). The second day was devoted to biomechanical assessments of user interactions with the prosthetic and haptic feedback system and collection of grip force data using an instrumented cylinder.

A sample of able-bodied participants aged ≥ 18 years, and without prior experience with the force feedback CUFF the myoelectric SHP, was also recruited. Exclusion criteria were the same as for the group with limb loss, with the additional criteria that participants must be able to learn to effectively control myoelectric sensors within a one-hour timeframe. This additional constraint was included so that all interventions and assessments could be completed within a three-hour timeframe. A three-hour timeframe was chosen to allow for enough time to learn to control the SHP, complete the study tests, and allow for rests within the study session to avoid muscular fatigue. Participants were given the opportunity to familiarize themselves with the device in the first hour (approximately 35 minutes of myoelectric control training of the SHP without the CUFF, and approximately 25 minutes of training with both the SHP and CUFF). The remaining two hours were allocated for biomechanical assessments. If at the end of the first hour of familiarization the participant was unable to modulate grip force, testing with that participant was discontinued due to inadequate myoelectric control. Two male participants (aged 61 and 81) were not accrued after being recruited due to inadequate myoelectric control.

### Experimental protocol

The study protocol was approved by the Mayo Clinic Institutional Review Board (IRB), and all the participants gave written informed consent before enrollment in the study.

#### Familiarization

Once the SHP and socket or orthosis were donned by the participant, the gain settings for the myoelectric control were optimized. Participants of both cohorts familiarized themselves with control of the SHP (without the CUFF) for a period of 35 minutes. Familiarization included picking up and stacking objects including blocks and collapsible drinking cups, as well as picking up and placing down objects of different sizes, shapes, and materials. For the group with limb loss, additional familiarization (in addition to the 35 minutes) involved activities of daily living such as donning and doffing a shirt/sock, tying a shoe, writing, using scissors, picking up feeding utensils, turning paper, and picking up small objects such as paper clips and coins. Then a familiarization period with the addition of the force feedback CUFF occurred (approximately 25 minutes). Participants were instructed to focus on the sensation given by the CUFF during a range of grip forces from maximal grip to a very light grip.

During familiarization and testing, participants were encouraged to take self-timed breaks and were also required to rest if they were showing symptoms of fatigue. All training and data collection phases were conducted by a licensed physical therapist (MVS).

#### Experimental set-up

**Fig 3:**
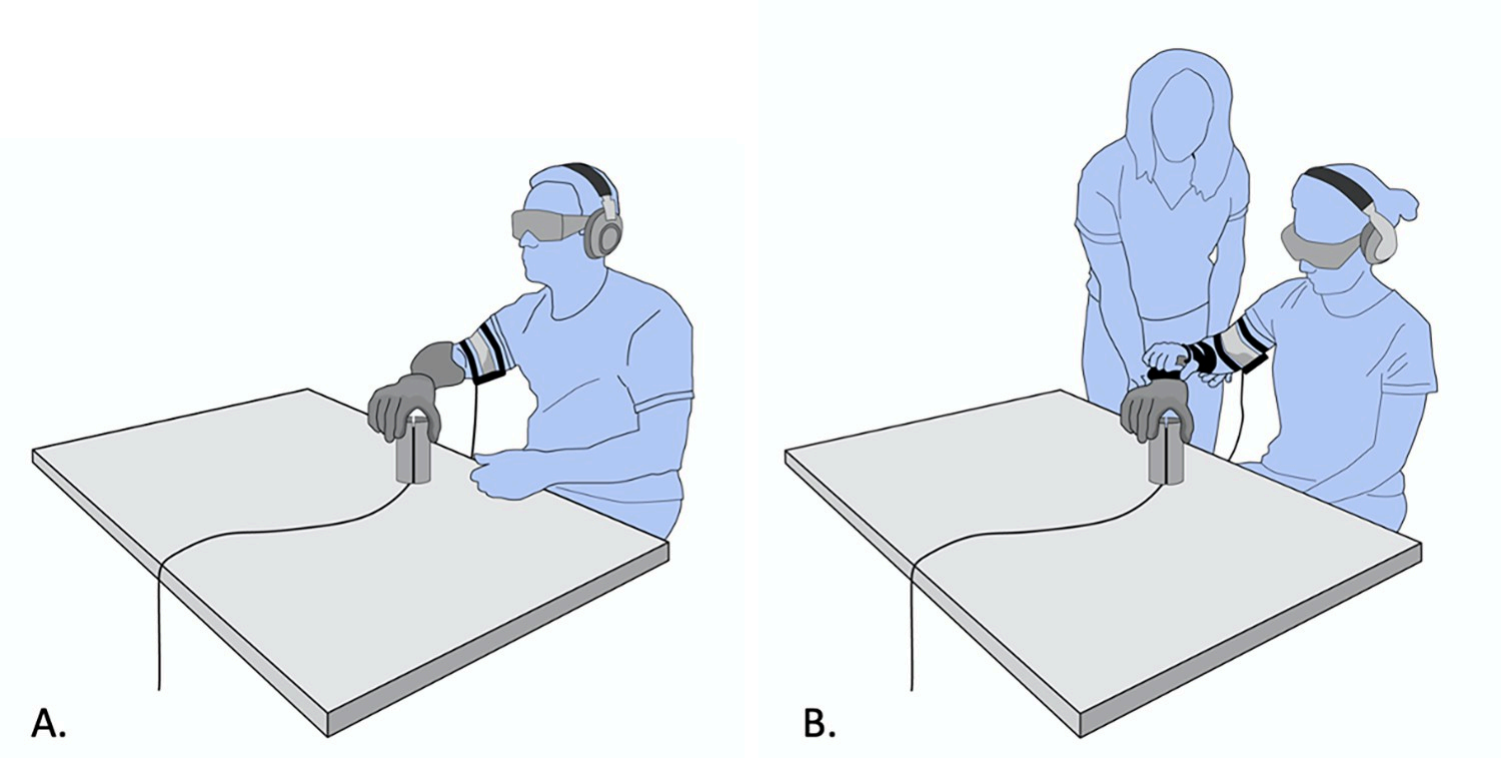
Experimental set-up. A) The experimental set-up used for the cohort with limb loss, and (B) the experimental set-up for the able-bodied cohort.

**Fig 4:**
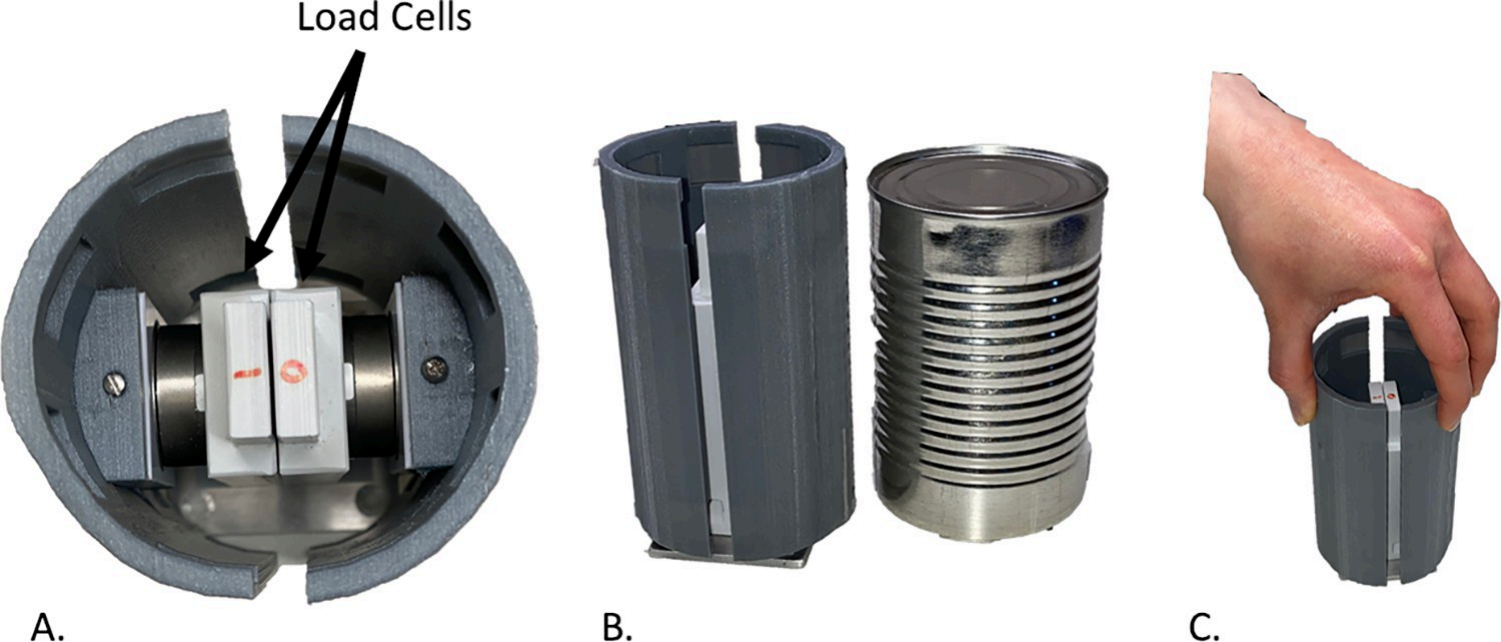
Instrumented cylinder. (A) From the left, view of the load cells from the top, (B) the cylinder next to a 400 g can, to understand the dimensions next to a functional object (b), and an example of the grasp executed during the experiments, with the index and middle finger contacting one hemisphere of the cylinder and the thumb contacting the other hemisphere.

The participants were comfortably seated at an adjustable height table with the CUFF placed on the upper arm, and the cylinder in front of them. It should be noted that due to the design of the CUFF, it could be placed on any cylindrical body part such as the forearm or upper arm. To be consistent and not interfere with myoelectric sensor placement, this study placed the CUFF on the upper arm. The calibration of the CUFF involved an auto-adjustment of the fabric belt around the participant’s arm in which the fabric was tightened until the two motors reached the stall condition and then release leaving the absorbed current close to zero. This process guarantees the complete contact of the belt on the arm. Therefore, the length of the belt is individualized to the anthropometric measurement of the participant’s arm, but the starting pressure (close to zero) on the arm is standardized between participants. The cylinder was affixed to the table at a self-selected distance from the table edge (ranging from 10–15 cm from the table edge), on the side of the arm being tested (Fig 3). For the able-bodied cohort, the testing arm was the dominant arm. For the group with limb loss, the testing arm was the side of limb loss. The cylinder was rotated so that one half of the cylinder (one hemicylindrical blade) was in contact with the SHP’s index and middle finger, and the other half was in contact with the opposing thumb during testing when gripping the cylinder from the top (Fig 4). The exact positioning of the cylinder was marked and repeated for both testing conditions (with and without CUFF feedback). A three-finger grasp was chosen to guarantee the best trial to trial repeatability. Due to the synergistic grasp and the shape of the instrumented object (cylindrical), other grasps caused the fourth and fifth fingers to slip off the cylinder during the grasp, rotating the cylinder, thus reducing trial to trial repeatability.

Able-bodied participants supported an additional 520g of mass distal to their native hand, which was the weight of the orthosis used to mount the SHP. In addition, the test required several minutes of consecutive static shoulder abduction, and the duration of the experimental protocol was 3 hours. Therefore, manual support was provided to the able-bodied participants’ forearms to prevent shoulder musculature fatigue and musculoskeletal overuse injury. A mobile arm support (i.e., gravity compensator) did not allow enough degrees of freedom to properly grip the cylinder with the SHP while still allowing for relaxation of the shoulder musculature; therefore, manual support was given by MVS. The physical therapist only provided support and reminders to allow her to support the weight of the arm; she did not provide any manual or verbal cues that would influence performance during testing. Manual forearm support was not provided to the participants with limb loss since they had to lift only the SHP which was similar in weight to their owned prostheses and the mass of the SHP did not extend beyond that of a native hand.

#### Training for the targeted force

Prior to data collection, participants were trained to grip with a targeted moderate amount of force (less than the maximal force generated by the SHP and more than the minimum grip required to activate the CUFF). This training provided the participants with an understanding of the stimuli provided by the force feedback CUFF related to the grip of the SHP.

1. *Quantifying the minimum grip force to activate the CUFF*. The participants were instructed to slowly squeeze the cylinder and stop squeezing the moment they felt the first stimulus exerted by the CUFF on the arm. The minimum force grasp was repeated three times and the applied force of the two loads cells was recorded. The median value of peak force applied in all the trials was defined as the minimum grip force.
2. *Quantifying the maximum grip force of the SHP*. The participants were instructed to squeeze the cylinder as hard as they could and hold the grip for 3 seconds. The maximal grasp was repeated three times and the peak of the force of the two load cells was recorded. The median of multiple trials was defined as the maximum grip force.
3. *Training to modulate grip force*. Once the participants were trained to sense the minimal and maximal stimulus of the CUFF, they were instructed to gradually squeeze the cylinder with the SHP to receive a moderate amount of stimulus on their arm, thus producing a moderate grasp force. Performing a moderate amount of grasp force (range) rather than a specific grasp magnitude (point) was the verbal cue since the SHP’s synergistic grasp creates a residual current that varies between trials (of the same participant) and this current tells the CUFF how much to squeeze. Based on the displacement of the CUFF fabric (caused by rotation of the motors), the examiners provided verbal feedback to indicate if the training trial was “too much” (when they reached the maximum force), “not enough” (when the grasp was not forceful enough to activate the CUFF), or “good” (when the grip was larger than minimum but less than maximum grip force). Following 10 trials for which the participant received the verbal feedback of “good”, the training was complete. The forces, registered by the load cell, were recorded during the training. Video was also recorded during training. Following data collection on a subsequent day, video was reviewed and the trials that were “good” were identified so that the median moderate grip force of the “good” trials could be calculated.

### Data collection

Headphones and non-prescriptive glasses were donned by the participants for data collection. The headphones played pink noise (https://mynoise.net/NoiseMachines/whiteNoiseGenerator.php) to eliminate auditory feedback about the grip produced by the actuators of the SHP and the CUFF. Pink noise delivers less intensity in the higher frequencies and more intensity in the lower frequencies, making it more comfortable for the human ear than white noise and still effective at blocking sound. The lenses of the glasses were modified to reduce vision so that participants could not determine the positioning of the fingers of the SHP around objects. A layer of thin, opaque foam was adhered to the lenses to obstruct participants’ view.

Prior to donning the headphones and glasses, participants were instructed to squeeze the cylinder with moderate force as they had done during training. They were told that two sets of five repetitions per condition would be completed with 2 minutes of rest between sets. During rest, the headphones and glasses were removed. Data from the instrumented cylinder was collected at 100 Hz.

During data collection, examiners noted, not surprisingly, that there were differences, among the able-bodied participants, in their ability to use a myoelectric prosthesis. Video of the data collection was reviewed on a subsequent day by MVS and the participants were subjectively categorized on a 1–3 scale of myoelectric control/skill. Participants were assigned a 3 if they consistently demonstrated the ability to contract wrist flexor muscles for SHP grasping and wrist extensors for SHP opening without co-contraction, a 2 if they began the data collection requiring moderate cuing to relax certain muscle groups in order to avoid co-contraction but improved over the course of the training, and a 1 if they required maximal cuing throughout the training and co-contraction was observed frequently even during testing.

#### Testing order

For the able-bodied participants, block randomization (in groups of six) of the testing order of condition (utilizing the CUFF versus not utilizing the CUFF) was performed to assure that equal number of participants performed each testing order. Table 2 shows the two possible testing orders. Ten participants used the CUFF first (6 male/4 female) and 9 participants used the CUFF last (2 male/7 female).

**Table 2:** The possible testing orders accomplished by the able-bodied participants during the experiment.

Testing order	First testing condition	Second testing condition	Third testing condition
1	SHP + CUFF	Sound or contralateral Limb	SHP
2	SHP	Sound or contralateral Limb	SHP + CUFF

The sound limb or the contralateral limb testing was done in between conditions with the SHP to provide musculoskeletal rest for the limb performing myoelectric control of the SHP.

For the participants with limb loss, the first participant began the test while not wearing the CUFF. The subsequent testing alternated between wearing the CUFF first and not wearing the CUFF first, etc. The exact positioning of the cylinder was marked between conditions so that the rotation of the cylinder could be standardized.

### Data analysis

The data recorded were analyzed with functional Principal Component Analysis (fPCA). Briefly, the fPCA is a statistical method that allows comparison between motions that occur in nature, such as human movement [30]. In human movement, variability between repetitions of the same task naturally exists. This variability is larger in humans with impairments such as hemiparesis due to stroke [31] and missing sensation due to limb loss. The goal of rehabilitative interventions is to reduce this variability which can be quantified with fPCA over the entire motion of the task. In this study, we used the fPCA to compare differences in precision, during a grasping motion, with or without the use of CUFF feedback.

To avoid the inclusion in this analysis of undesired features (an accidental grasp before the trial was started, or pressure on the load cells due to resting the hand with a small amount of pressure on the top of the cylinder before grasping), due to misalignments in time, the following pre-processing procedure was performed to all the data recorded from instrumented cylinder task:

1. The signal was manually segmented to remove the portion of the recording in which the participant was not actively performing the task. 50 frames before and after each repetition were included.
2. A resample of the signal was executed to have a standardized length of 400 samples for each trial.
3. A time warping technique synchronized (in time) all the signals of the data. This procedure, known in the literature as Dynamic Time Warping (DTW), identifies the optimal time-shift and time-stretch of one signal with regard to another. DTW, described in detail by Averta et al [30], was necessary to mediate the ten repetitions and to avoid misalignment in this data.

In this work, we quantified differences in precision of force during an entire grasping motion, with or without the use of CUFF feedback. The main hypothesis was that with the stimuli provided by the CUFF, the subjects would be able to grasp in a more natural way (similar to the sound limb testing condition), compared to the no feedback condition. Aligned with this hypothesis, given a specific number of fPCs used to reconstruct the signal, the explained variance would be lowest in condition of no CUFF feedback.

If the first functional component achieves 100% of the explained variance (of the grasping force motion), then the grasp would be considered perfectly precise. However, human movement is never perfectly precise and involves corrections during a motion as well as variance between repetitions. fPCA allows us to speculate that if the explained variance is high there were few or no corrections in the grasp action, and that the participant was able to grasp with similar force patterns from trial to trial. If there is the necessity to add more components to explain the signal, then there are likely more corrective actions to obtain the targeted force and more variability between trials.

## Results

As expected, and for both cohorts, the sound limb condition resulted in higher first functional principal components (95%—participants with limb loss; 89%—able-bodied cohort) than any condition with the SHP.

**Fig 5:**
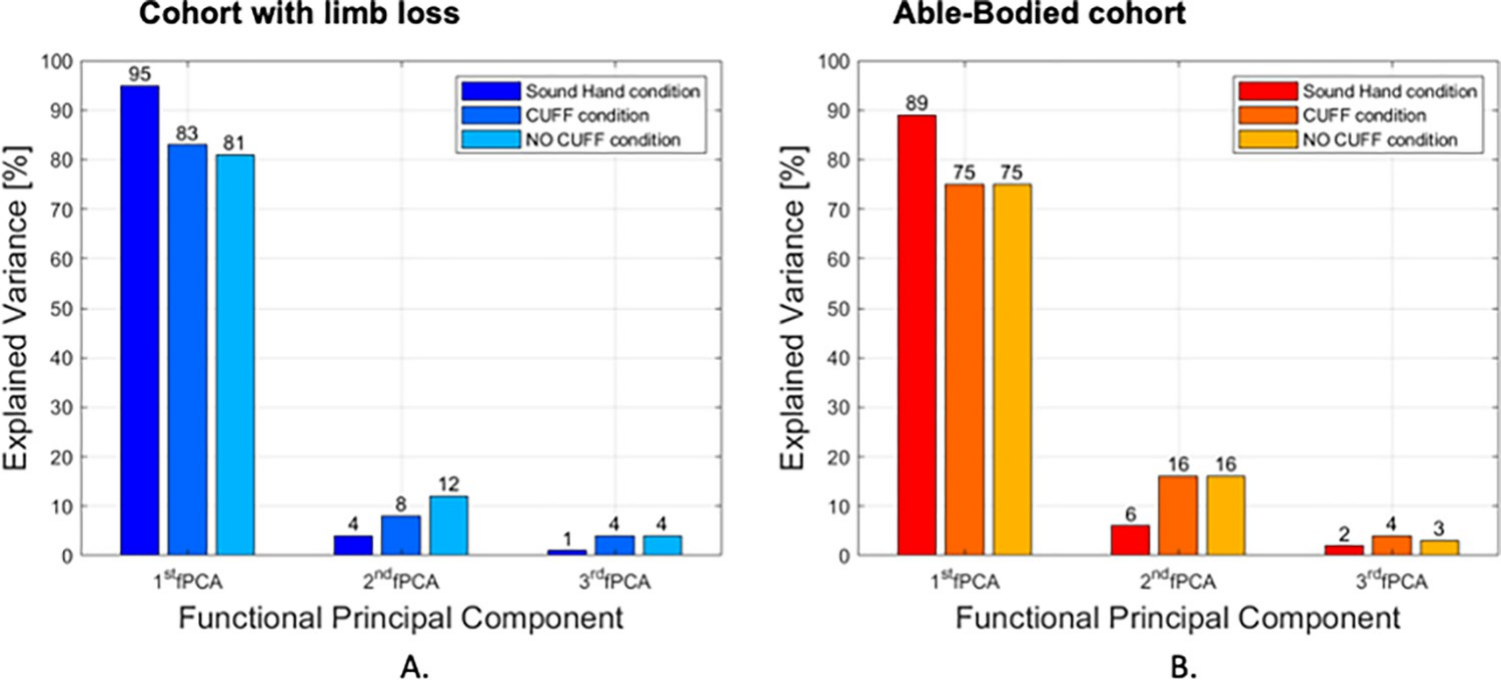
Explained variance of the entire population. Explain variance of the first three fPCA for the entire cohort of the participant with limb loss (on the left), and able-bodied participants (on the right).

For the participants with limb loss, use of the CUFF resulted in the first functional principal component explaining 83% of the variability compared to 81% without the CUFF (Fig 5). Because participants used different types of prostheses in their daily life (Table 1), the limb loss participants (N = 5) were categorized into two subgroups: body-powered (N = 2) and myoelectric prosthesis users (N = 3).

**Fig 6:**
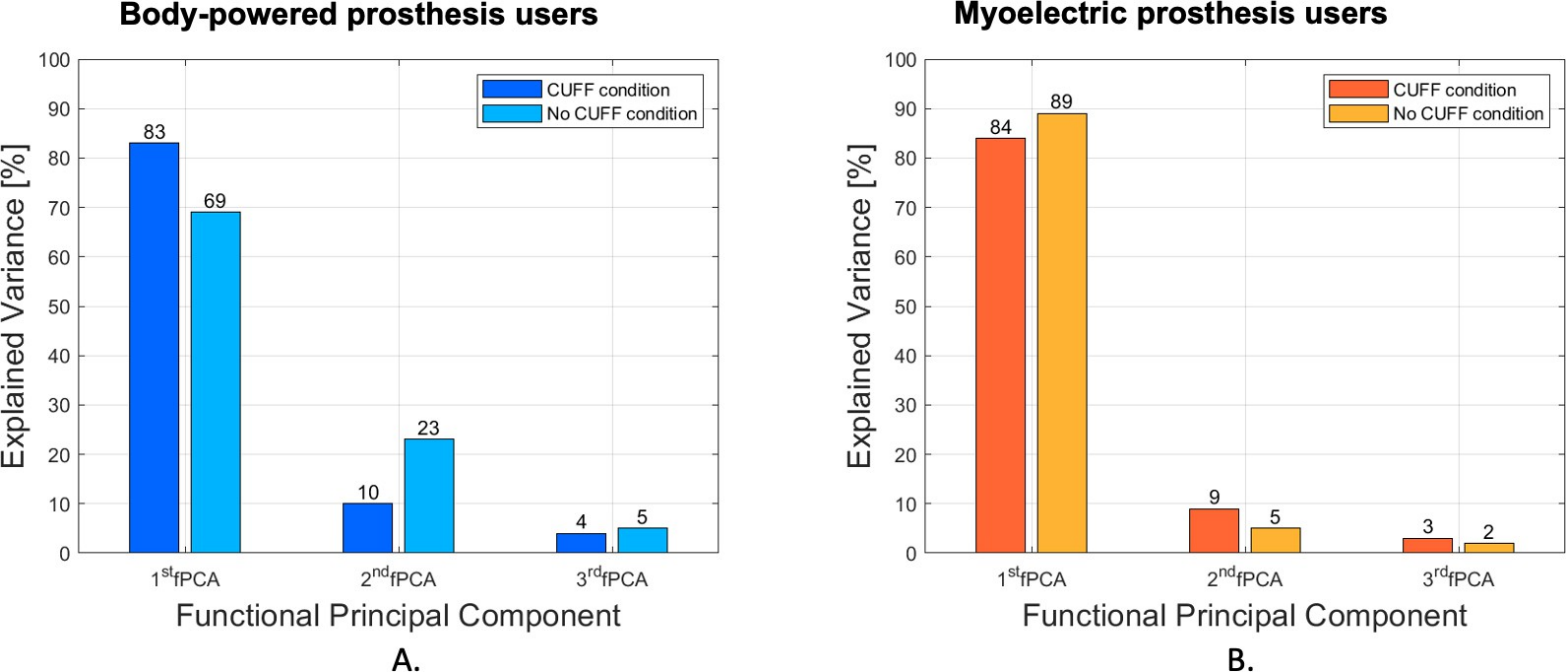
Explained variance of the sub-group of the limb-loss cohort. Explained variance of the first three principal components for the sub-group of body-powered prosthesis users (on the left), and the sub-group of the myoelectric prosthesis users (on the right).

Analysis by subgroup indicated that the participants who regularly used a body-powered prosthesis, when using the CUFF, reached 83% of explained variance compared to 69% of explained variance when the CUFF was not used. However, experienced users of myoelectric prostheses had percentages of explained variance of 84.5% and 89% with and without the CUFF, respectively (Fig 6).

**Fig 7:**
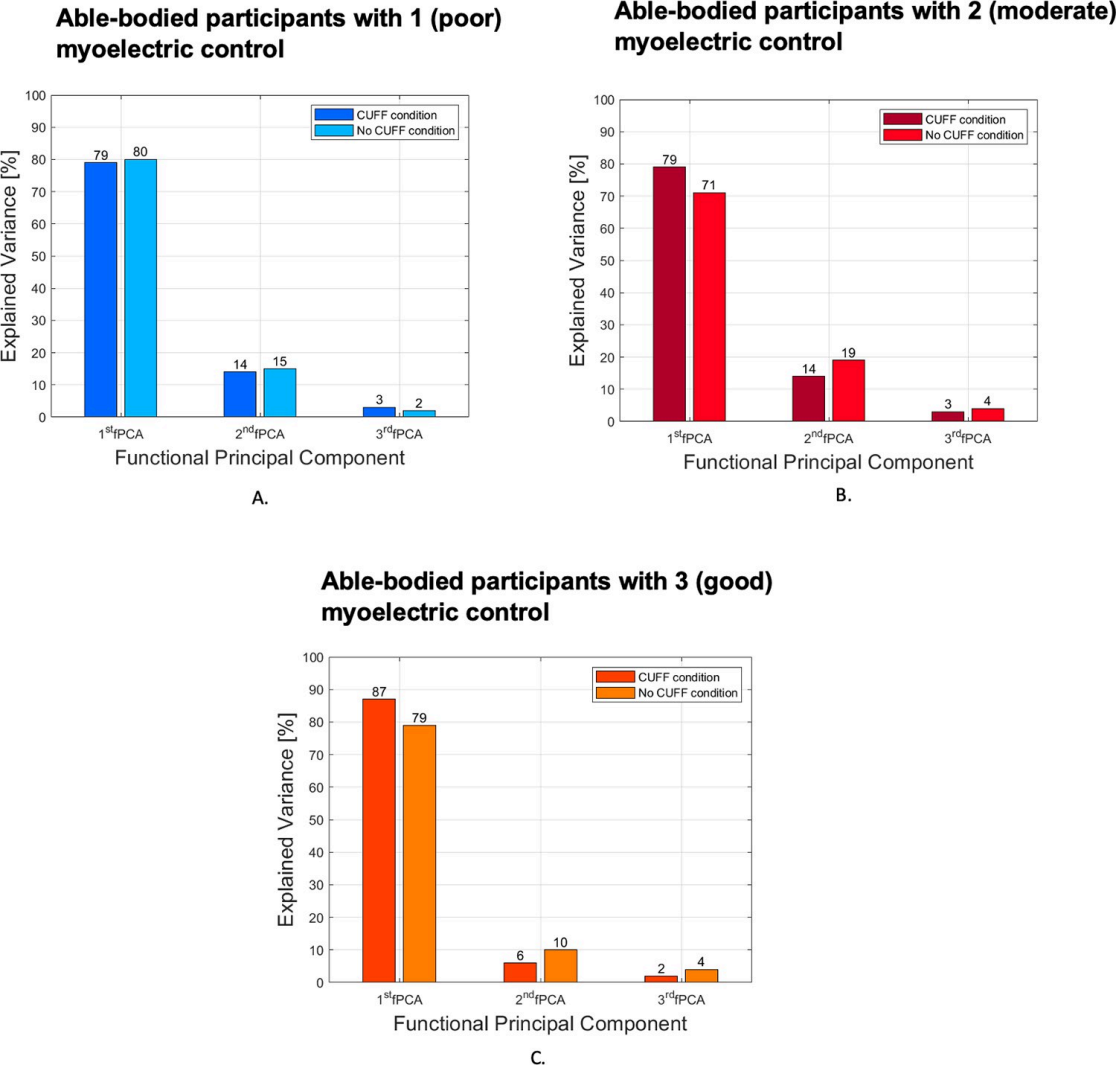
Explained variance for the sub-group of the able-bodied participants by subjective rating of myoelectric control skill. On the top left are the results of the sub-group with “poor” myoelectric control who required maximal cues to minimize co-contraction of their wrist muscles. On the top right are the results of the sub-group that needed “moderate” cues but improved during training; and on the bottom the sub-group that demonstrated “good” myoelectric control without co-contraction of the wrist muscles.

For the able-bodied cohort, there was no substantial difference in percentage of explained variance between the two conditions with and without the CUFF (Fig 5). However, the sub-group of five participants, who were subjectively rated to have poor myoelectric control skills (score of 1), obtained a percentage of explained variance of 79% and 80% for the condition with and without the CUFF, respectively. For the moderate myoelectric control skills (score of 2) subgroup, the explained variance was 79% with the CUFF and 71% without the CUFF. For the good myoelectric control skills (score of 3) subgroup, the explained variance was 87% with the CUFF compared to 79% without the CUFF (Fig 7).

**Fig 8:**
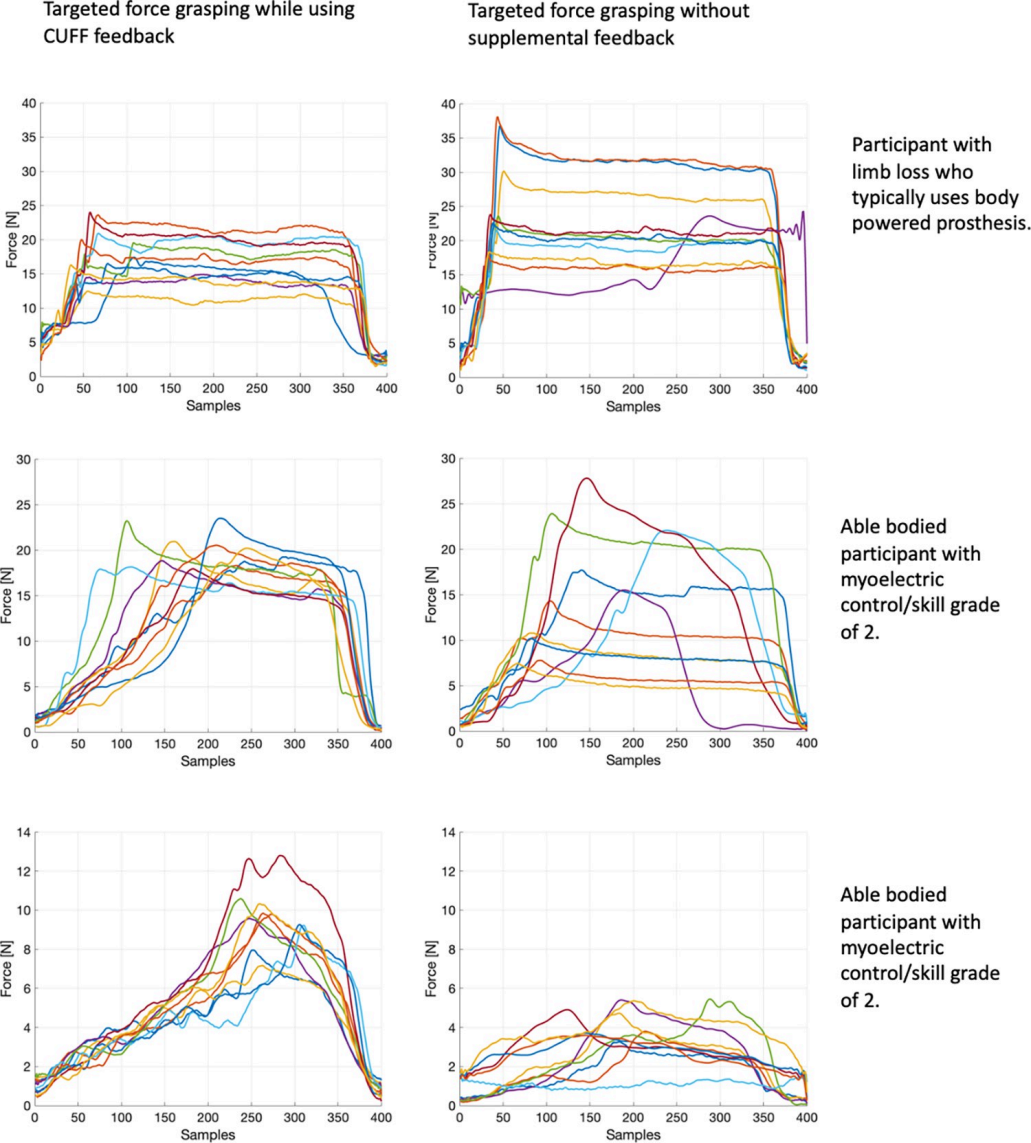
Individual forces curves for one participant with limb loss and two able-bodied participants. Participants completed 10 trials of a targeted force grasp with CUFF feedback (left column) and without supplemental feedback (right column). The top row is the force curves from a participant with limb loss who typically uses a body powered prosthesis. The bottom two rows are force curves from two able-bodied participants who were subjectively graded as a 2 for the myoelectric control/skill category. Each trial on the graphs represents a full grasping cycle.

To assist with the interpretability of the fPCA, Fig 8 is provided which depicts individual force curve data for three participants; a participant with limb loss who typically uses a body powered prosthesis and two able-bodied participants who began the study requiring moderate cuing to relax certain muscle groups in order to avoid co-contraction but improved over the course of the training (myoelectric control/skill grade of 2). In these three examples, the trials with the feedback from the CUFF had visually less variability between trials. This can be seen in the magnitude of the force as well as the shape of the force curves.

## Discussion

This study tested the ability of the CUFF, which provided SHP grip force feedback, to improve the participants’ grasp force control. To add context to these findings, the grasp of the sound limb was also tested, and as expected, was shown to have more grasping precision than the SHP. The precision of the grasping motion (quantified with fPCA) did not differ between the CUFF and no CUFF conditions for either those with limb loss or the able-bodied cohort. Although grasping precision differences were not seen when the cohorts were analyzed as a whole, the subgroup of participants with limb loss who regularly use body powered prostheses and the subgroups of able-bodied participants who demonstrated moderate and poor skill of myoelectric control demonstrated improved precision of the grasping motion (as measured by fPCA) when the CUFF was used. Participants of both cohorts had better grasp precision with their sound limb (as measured by fPCA), as expected.

In Averta et al. the input kinematic data were analyzed with the fPCA to investigate upper limb movement following stroke [31], other examples of using fPCA to investigate upper limb motions have not been found in the literature. This study’s approach of inputting force data during a grasping task into fPCA adds to the limited body of work utilizing fPCA to compare upper limb movement patterns. FPCA appears to be a promising tool to differentiate groups of individuals who have varied prosthetic experience, coordination, or myoelectric control.

According with the results, for participants with limb loss who had the least amount of experience using myoelectric control (body powered prosthesis users), the force feedback seemed to improve grasping precision. A plausible explanation for this is that humans develop internal models through experiences and these internal models allow them to function efficiently using feed forward approaches [15]. In a recent review of the literature, Sensinger and Dosen [15] present a comprehensive overview of motor learning principles related to both natural and supplemental closed feedback loops; they propose how this knowledge should guide data interpretation and future research and development related to upper limb prostheses. Sensinger and Dosen state that “when subjects have not yet received extensive training, (supplemental) feedback is useful–both to develop internal models as well as execute real-time corrections. Over time, however, as participants develop better internal models, the usefulness of (supplemental) feedback for real-time corrections may fade”. Training allows humans to perceive and interpret incidental sources of information. The prosthetic user receives vibratory and auditory cues from the motor. Prosthetic users become highly reliant on the high-fidelity visual information of the prosthetic hand’s movement. Training also allows users to understand the graded output of their forearm muscle contractions. While our study design reduced the visual and auditory information about the prosthetic hand, participants were still able to benefit from vibratory cues caused by the motor and internal models about muscle contractions. Likely strong internal models and use of incidental feedback allowed the myoelectric prosthetic users to perform well even without the use of the CUFF. There was also no benefit to using the CUFF for the sub-set of able-bodied participants who were subjectively graded as a 1 (indicating that throughout the course of the experiment they had poor myoelectric control). Our able-bodied participants had a very short time to familiarize themselves with myoelectric control and this sub-set of individuals may have needed more time to develop coordination of the forearm muscles before the effect of the force feedback could be tested. In summary, the value of supplemental feedback is reduced when already-available incidental feedback exists along with a well-developed internal model.

While it is important not to translate findings from the able-bodied cohort into clinical applications for those with limb loss, able-bodied cohorts with simulated functional limitations are sometimes studied when sampling with proper power would be too time and resource intensive [32, 33]. Findings from an able-bodied cohort can then help refine research questions for future clinical trials for persons with limb loss, such as the work done by Fu et al. who demonstrated that inter-limb training improved participants’ ability to grasp a targeted force [33]. It is possible that the able-bodied cohort in this study was representative of patients with a relatively new limb loss condition since both are naïve to using a prosthesis. If grasp force feedback for the SHP does improve precision in persons with new limb loss, then technology such as the CUFF would have value in rehabilitation settings. Findings that the CUFF benefited persons with limb loss who have less myoelectric control experience support the results from the able-bodied cohort. The CUFF may assist myoelectric control users of the SHP become proficient more quickly. However, the sample size of participants with limb loss was small, therefore interpretations from these data should be made cautiously, and future studies should have larger sample sizes of persons living with limb loss. Likewise, future research should investigate whether current grasp force feedback technologies are useful only during early learning stages or whether there is a long-term benefit and therefore should be integrated into sockets.

One of the limitations of this study design was that the task of grasping the top of the cylinder did not also include lifting the cylinder. A large majority of functional tasks involve a grasp and a lift, and therefore the study task was not functional. Additionally, in the real-world setting, individuals would not have had vision and hearing blocked, therefore, our approach was so constrained that it does not translate to the natural free-living environment. These methods were chosen because the unique design of the SHP (single tendon, flexion synergy, single motor) requires an incremental investigation of supplementary feedback that starts with demonstration that the provided feedback interface is effective in transmitting the desired grasp force information [15]. The next step in designing and testing supplementary feedback for the SHP will require addressing the limitations of the current study; 1) measuring whether supplementary feedback benefits the user even with incidental feedback of vision and hearing, 2) testing tasks that are more representative of functional activities, and 3) allowing participants to use feedback over a longer time period and in the home setting as is currently being done by others [19]. Further, since the CUFF feedback demonstrated improved precision during grasping in subgroups of our two cohorts, understanding if supplemental feedback is beneficial to accelerate mastery of myoelectric control or with certain patient sub-groups is also needed.

## Conclusion

This study demonstrated that the supplementary force feedback provided by the CUFF was beneficial to modulating the grasp force of the SHP with precision, however the in-lab testing protocol may not translate to the real-world and the CUFF may not benefit those who already have extensive experience with myoelectric control. Future development of supplementary force feedback for the SHP should include functional tasks in free-living environment, no restrictions to incidental feedback that is available in the real world, and longer-term use of the supplemental feedback.
